# Fluoroless Catheter Ablation of Cardiac Arrhythmias: Change Is Inevitable

**DOI:** 10.19102/icrm.2020.110406

**Published:** 2020-04-15

**Authors:** Mansour Razminia, Paul Zei

**Affiliations:** ^1^AMITA Health St. Joseph Hospital, Elgin, IL, USA; ^2^Brigham and Women’s Hospital, Harvard Medical School, Boston, MA, USA; ^3^Harvard Medical School, Boston, MA, USA

**Keywords:** Catheter ablation, fluoroscopy, near-zero, radiation

Historically, fluoroscopy has been an essential tool to facilitate the eradication of cardiac arrhythmias with catheter ablation. However, experience has also shown fluoroscopy to be associated with a number of radiation-induced morbidities, including skin injury, cataract, and malignancies, among others.^1–6^ Fatal cases of malignancy, for example, have been assessed to occur at rates of 0.7 to 1.4 per 1,000 women and 1.0 to 2.6 per 1,000 men following 50 to 60 minutes of fluoroscopy over the course of catheter ablation procedures.^7,8^ Other studies have illustrated the significant burden of orthopedic injuries resulting from the regular use of lead protective apparel.^9^ As an increasing number of patients undergo ablation procedures, efforts to significantly reduce or—ideally—eliminate radiation exposure in patients, operators, and laboratory staff are of paramount importance.

Understanding the significant direct and indirect risks related to radiation, a number of electrophysiologists have committed to refining current techniques to reduce and eliminate radiation exposure while maintaining the highest standards of procedural safety and efficacy. An ever-growing body of literature has documented these efforts toward performing ablation procedures without fluoroscopy, to the point that, we feel—with few exceptions—a fluoroless approach should be the new technical standard adopted in ablation procedures.

In this issue of *The Journal of Innovations in Cardiac Rhythm Management*, Huang et al. report the first case of a near-zero fluoroscopic approach for laser balloon pulmonary vein isolation (PVI) ablation.^10^ The authors relied on intracardiac echocardiography and direct endoscopic visualization of the pulmonary veins and other anatomic structures to minimize fluoroscopy. Brief fluoroscopy (0.3 minutes) was used during transseptal puncture to confirm that the tip of the transseptal needle did not extend beyond the dilator prior to dragging the transseptal assembly from the superior vena cava to the fossa ovalis. Another brief fluoroscopy dose (0.2 minutes) was used to confirm that the laser balloon tip was beyond the sheath prior to maneuvering the assembly from the left inferior pulmonary vein to the right inferior pulmonary vein and, hence, avoid any potential damage to the posterior wall. The total fluoroscopy time used during this case was 0.5 minutes, which was considerably lower relative to the mean fluoroscopy time reported in a multicenter randomized pivotal trial comparing laser balloon PVI and radiofrequency PVI (35.6 ± 18.2 minutes).^11^

This case report shows the feasibility of performing successful near-zero fluoroscopic laser balloon PVI without complications. It also illustrates the continuous effort of the electrophysiology community to minimize or eliminate fluoroscopy use in electrophysiology procedures. Since Reddy et al. reported on the catheter ablation of atrial fibrillation without fluoroscopy in 2010,^12^ there have been numerous reports on the safety and efficacy of fluoroless catheter ablation for all types of arrhythmia, including atrial fibrillation, atrial tachycardia, and ventricular tachycardia.^13–16^ Special situations in which a fluoroless technique would not be recommended as an initial approach include ablation in the epicardial space and in patients with complex congenital heart disease.

As the evidence supporting the benefits of a fluoroless approach grows in the face of diverse patient cohorts, arrhythmia types, and ablation technologies and platforms, arising both in the academic and private sectors, some may wonder not *if* but *when* this approach will be adopted by the vast majority in the electrophysiology community. This is especially the case when considering the benefits of fluoroless ablation not only for patients and operators but also the vital community of allied health professionals present in the laboratory, without whom we would not be able to provide optimal care as electrophysiologists. A large multicenter, prospective, randomized clinical trial comparing fluoroless to traditional fluoroscopically guided ablation may serve to eliminate lingering doubts about the safety and efficacy of the fluoroless approach. However, among the growing community of electrophysiologists who have transitioned to a completely fluoroless approach to ablation, an ethical challenge may be discerned with respect to exposing patients and laboratory staff to radiation unnecessarily in contradiction to the mantra of “as low as reasonably achievable.”

Aside from the practicality of such a trial, an additional factor influencing operator comfort with “going fluoroless” is the associated learning curve required to transition from traditional techniques learned over years of practice to a different approach. We advocate a stepwise approach to incorporating fluoroless techniques be adopted into one’s practice, which is predicated on a commitment to increase reliance on the wealth of information gleaned with intracardiac echocardiography (ICE) and electroanatomical mapping (EAM) systems. Practically speaking, this can be slowly achieved by starting with relatively simpler procedures and challenging oneself to minimize and, eventually, eliminate fluoroscopy use while substituting ICE and EAM data for guidance. Importantly, as the community of electrophysiologists committed to operating fully fluoroless laboratories grows, there is an ever-increasing and highly supportive community of colleagues willing and able to offer guidance and resources to help newcomers in their journey to becoming fluoroless operators.

Beyond informal mentorship and support, efforts are also underway to organize the community of electrophysiologists dedicated to exploring fluoroscopy reduction techniques and capitalize on the momentum gained over the past decade. In this vein, a Fluoroscopy Reduction in the Electrophysiology Lab Council was established in 2019, composed of thought leaders in this space whose primary aim is to highlight gaps in practice and identify unmet clinical needs with respect to fluoroscopy reduction and allied concepts. One of the Council’s primary objectives is to develop educational resources and programming to meet the needs of the broader electrophysiology community, whether individuals are merely considering the merits of adopting fluoroless techniques or are fully invested in transitioning to a fluoroless laboratory environment. The ultimate goal of these efforts is to improve the penetration of these techniques into contemporary electrophysiology practice. By extension, it is our sincere hope that, as fluoroless ablation gains a stronger foothold within the electrophysiology community—particularly within academic medical centers training the next generation of electrophysiologists—current and future fellows may begin incorporating these techniques and experiencing their benefits even as they are completing their training, thus emerging already ready to deploy this approach in their own practice. As this accompanying publication illustrates, this can be achieved beyond the standard radiofrequency or cryothermy approaches.

The preponderance of evidence and clinical experience with fluoroless ablation speaks to its enhanced safety for patients, electrophysiologists, and laboratory staff alike.
